# Complete genome sequence of *Proteiniborus* sp. MB09-C3, isolated from the feces of the black soldier fly larvae

**DOI:** 10.1128/MRA.00608-23

**Published:** 2023-10-17

**Authors:** Wanling Qiu, Junyu Zhu, Chao-Jen Shih, Yen-Chi Wu, Shu-Jung Lai, Yi-Ting You, Chih-Hung Wu, Ching-Hua Liao, Haozhe Lin, Shilong Li, Yujia Yan, Xuming Luo, Yin Li, Sheng-Chung Chen

**Affiliations:** 1 School of Resources and Chemical Engineering, Sanming University, Sanming City, Fujian, China; 2 College of Environment and Safety Engineering, Fuzhou University, Fuzhou, Fujian, China; 3 Bioresource Collection and Research Center, Food Industry Research and Development Institute, Hsinchu, Taiwan, Republic of China; 4 Graduate Institute of Biomedical Sciences, China Medical University, Taichung City, Taiwan, Republic of China; 5 Research Center for Cancer Biology, China Medical University, Taichung City, Taiwan, Republic of China; 6 Department of Life Sciences, National Chung Hsing University, Taichung City, Taiwan, Republic of China; 7 Fujian Provincial Key Laboratory of Resources and Environmental Monitoring and Sustainable Management and Utilization, Sanming University, Sanming, Fujian, China; University of Maryland School of Medicine, Baltimore, Maryland, USA

**Keywords:** *Proteiniborus*, black soldier fly

## Abstract

Here, we report the complete genome sequence of *Proteiniborus* sp. MB09-C3 (= BCRC 81405), isolated from the feces of black soldier fly (*Hermetia illucens*) larvae. The genome of strain MB09-C3 was selected for further species delineation and comparative genomic analysis.

## ANNOUNCEMENT

Strain MB09-C3 was isolated from the feces of *Hermetia illucens* larvae, commonly known as black soldier flies. The black soldier fly eggs were purchased from a shop in Taobao in June 2020 and subsequently raised by a colleague at our university. The larvae were fed wheat flakes and kept indoors in a plastic container with a transparent cover at room temperature (~25°C). The feces sample was collected and inoculated into MB/W media (1, 2) using a sterile spoon on 30 May 2021. Strain MB09-C3 was further isolated and identified using the process combined with serial dilution and rolling-tube technique (3) as well as 16S rRNA gene clone sequencing with 8F (5′-AGAGTTTGATCCTGGCTCAG-3′) and 1492RU (5′-TTTTAATTAAGGTTACCTTGTTACGACTT-3′) primers (4) for three rounds to obtain a pure isolate. The purity of this strain was verified by observation of morphology, 16S rRNA gene, and genome sequencing. Based on the results of BLASTN analysis (5), strain MB09-C3 showed highest similarities to one characterized and validly strain, *Proteiniborus indolifex* BA2-13^T^ (6) (97.35%), which indicated that strain MB09-C3 could be a novel species in genus *Proteiniborus*.

The strain MB09-C3 has been deposited in the Bioresource Collection and Research Center, Taiwan as strain BCRC 81405. It was grown in the MB/W media prepared according to our previous publications (1, 2) and incubated at 30°C. The genomic DNA of strain MB09-C3 was isolated and purified using the NucleoBond HMW DNA kit (Macherey-Nagel, Germany), according to the manufacturer’s instruction. The whole-genome sequencing was performed using the DNBSEQ-T7 platform (MGI Tech Co., Ltd.) and MinION sequencer (Oxford Nanopore Technology) at the Sangon Biotech (Shanghai) Co., Ltd.

For DNBSEQ-T7 platform, sheared genomic DNA fragments of approximately 300 bp were utilized to prepare a 150-bp paired-end DNA library. The library preparation was performed using Hieff NGS MaxUp II DNA Library Prep Kit for Illumina [Yeasen Biotechnology (Shanghai) Co., Ltd.]. The constructed library was sequenced using MGISEQ-2000RS High-throughput Sequencing Set (PE150 format), and 11,388,432 reads were generated and trimmed using Trimmomatic v0.36 (7). For MinION sequencer, sheared DNA fragments were straightly end-repaired, 3′ adenylated, and then ligated to adaptors pre-loaded with motor proteins. The product was purified using Agencourt AMPure XP Beads (Beckman, A63881). Finally, fragments larger than 1 Kb were screened for single-molecule nanopore DNA sequencing using Multiplex Ligation Sequencing Kit (SQK-MLK111.96-XL) on MinION Flow Cell (ONT, R9.4.1). Basecalling was performed using Guppy version 6.4.6 with default model (ONT). A total of 923,629 reads were obtained, followed by trimming using Porechop v0.2.4 (8), and filtering using NanoFilt v2.8.0 (9). DNBSEQ-T7 and MinION reads were hybrid *de novo* assembled using NextDenovo v2.5.2 (10). The assembly *N*_50_ value is 3,881,360 bp. The hybrid sequencing protocol generated ~1,032× mean coverage of the genome. Gene predictions and annotations were performed using NCBI Prokaryotic Genome Annotation Pipeline v6.5 (11).

The assembly yielded a 3,881,360 bp contig with 33.09% GC content. It was circularized by aligning and removing overlapping sequences at one end without rotation and predicted 3,779 genes. Default parameters were used for all bioinformatic analyses.

## Data Availability

The genome sequence of strain MB09-C3 has been deposited in GenBank under accession number CP127161. The version of the genome described in this paper is first version. The BioProject and BioSample accession numbers are PRJNA976962 and SAMN35447607. DNBSEQ-T7 and MinION raw reads were deposited in the Sequence Read Archive (SRA) under accession numbers SRR24759074 and SRR24759075, respectively. The 16S rRNA gene sequence of strain MB09-C3 has been deposited in GenBank under the accession number OR230128.
